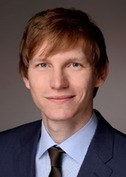# Enantioselective Desymmetrization of Cyclobutanones: A Speedway to Molecular Complexity

**DOI:** 10.1002/anie.201910767

**Published:** 2020-02-19

**Authors:** Jan Sietmann, Johannes M. Wahl

**Affiliations:** ^1^ Westfälische Wilhelms-Universität Münster Institute of Organic Chemistry Corrensstrasse 40 48149 Münster Germany

**Keywords:** C−C bond activation, cyclobutanone, desymmetrization, quaternary stereocenters

## Abstract

Cyclobutanones hold a privileged role in enantioselective desymmetrization because their inherent ring strain allows for a variety of unusual reactions to occur. Current strategies include α‐functionalization, rearrangement, and C−C bond activation to directly convert cyclobutanones into a wide range of enantiomerically enriched compounds, including many biologically significant scaffolds. This Minireview provides an overview of state‐of‐the‐art methods that generate complexity from prochiral cyclobutanones in a single operation.

## Graphical abstract

**Take the strain**: The ring strain of cyclobutanones makes them ideal for enantioselective desymmetrization. α‐Functionalization, rearrangement, and C−C bond activation can be used to directly convert cyclobutanones into enantiomerically enriched compounds, including many biologically significant scaffolds. This Minireview provides an overview of state‐of‐the‐art methods that generate complexity from prochiral cyclobutanones in a single operation.

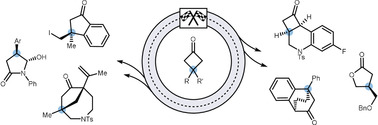

## 1 Introduction

**Scheme 1:**
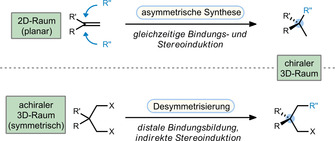
Establishing point chirality through asymmetric synthesis and desymmetrization of a prochiral molecule.

Chirality not only adds a new dimension to the complexity of molecular frameworks, but it also provides a handle for interaction with large structural networks, which are found in all living organisms.^[1]^ The asymmetric introduction of new stereocenters is considered one of the most stimulating challenges in organic chemistry, and has a great impact on related fields such as medicine and biology.^[2]^ Point chirality, the most prominent element of chirality, is traditionally introduced by energetically delineating an addition to two prochiral faces of a planar entity (Scheme 1).[^3^, ^4^] In an alternative approach, a symmetric three‐dimensional structure can be desymmetrized to establish a new stereocenter.^[5]^ In terms of synthetic applicability, desymmetrization offers several advantages, as the type of reaction and the nature of the stereocenter are not interconnected and sterically congested stereocenters can be introduced at a distal, sterically accessible position of the molecule.

With these specific advantages in mind, desymmetrization of cyclobutanones offers a range of attractive features for method development.^[6]^ For example, the ring strain^[7]^ enables unusual and otherwise difficult reaction trajectories, including polymerizations.^[8]^ Ring expansions are energetically downhill processes, and the corresponding five‐ and six‐membered rings are among the most prominent ring sizes found in nature. In addition, an exergonic energy profile provides several possibilities for the design of cascade reactions and the generation of multiple stereocenters in a single transformation.^[9]^

This vast build‐up of molecular complexity makes cyclobutanone desymmetrization an ideal tool for the synthesis of natural products.[^5b^, ^5e^] Moreover, highly congested positions are addressable, including quaternary stereocenters.^[5c]^ This Minireview is intended to highlight recent developments and stimulate synthetic applications of this powerful reaction ensemble.

## 2 α‐Functionalization

**Scheme 2:**
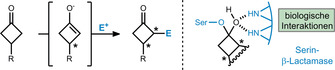
Left: Cyclobutanone desymmetrization by α‐functionalization. E=electrophile. Right: Mode of action of a cyclobutanone as a serine β‐lactamase inhibitor.

A viable method for cyclobutanone desymmetrization is α‐functionalization with an appropriate electrophile via formation of an initial enolate (Scheme 2, left). Such an endeavor leaves the core cyclobutanone motif intact and sets two consecutive stereocenters within the ring. Critical for reaction design is the high reactivity of the ketone function,^[10]^ which poses an unusual challenge when compared to simple ketone functionalizations.

As a consequence of the inherent strain and the precise arrangement of substituents, cyclobutanones tend to form hemiketals, which are key for their activity as β‐lactamase inhibitors, as studied by Dmitrienko and co‐workers in 2008 (Scheme 2, right).[^11^, ^12^] These predictable interactions with known biological residues provide further incentive for method development for the enantioselective synthesis of cyclobutanones in the future.

### 2.1 Aldol Reaction

**Scheme 3:**
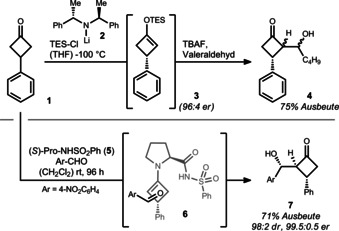
Top: The enantioselective deprotonation strategy of Honda. Bottom: The organocatalyzed approach of Aitken and Frongia.

Honda et al. conducted pioneering work related to enantioselective cyclobutanone desymmetrization through aldol reactions in the early 1990s (Scheme 3, top).^[13]^ Their strategy was based on an enantioselective deprotonation of 3‐phenylcyclobutanone (**1**) using chiral lithium amide **2** at low temperatures. The chiral enolate was trapped with triethylsilyl (TES) chloride to give silyl enol ether **3** in an enantiomeric ratio (er) of 96:4. The addition of valeraldehyde and tetrabutylammonium fluoride (TBAF) led to the formation of aldol **4** as a mixture of diastereoisomers.

In 2012, the groups of Aitken and Frongia developed an organocatalyzed desymmetrization of the same cyclobutanone **1** using proline‐based catalyst **5** to tackle previous issues regarding diastereoselectivity and cryogenic temperatures (Scheme 3, bottom).^[14]^ Catalyst **5** has a dual role: it activates cyclobutanone **1** by formation of a chiral enamine, and orchestrates the approach of the aldehyde through hydrogen bonding (**6** shows the preferred approach). Thus, good control of the diastereo‐ and enantioselectivity was achieved for activated aromatic aldehydes such as 4‐nitrobenzaldehyde. Product **7**, containing three contiguous stereocenters, was isolated in 71 % yield, thus highlighting the fast assembly of complexity with this method. Later, the same groups were able to extend the scope to nitrostyrenes by switching to a different type of organocatalyst (not shown).^[15]^

### 2.2 α‐Arylation

Based on seminal work by the groups of Jia^[16]^ and Britton,^[17]^ Lu and co‐workers initiated a study towards the desymmetrization of cyclobutanones through intramolecular α‐arylation.^[18]^ This strategy relies on synergistic palladium/enamine activation, which conveniently provides two handles for control of the enantioselectivity.

**Scheme 4:**
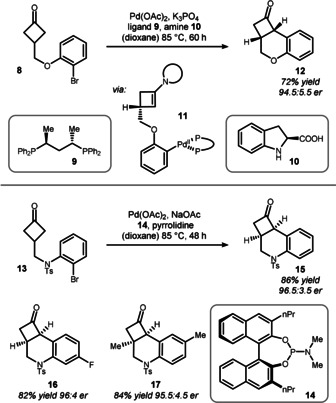
Synergistic palladium/enamine catalysis used by Lu. Ts=*p*‐toluenesulfonyl.

Cyclobutanone **8** with a pendent *ortho*‐bromoaryl group was treated with chiral phosphine **9** and chiral amine **10** under palladium catalysis (Scheme 4, top). The necessity of both chiral sources provides credibility for an intermediate such as enamine **11**. For the final C−C bond formation to afford tricyclic cyclobutanone **12**, the authors propose a Heck‐type mechanism.

Interestingly, for starting materials bearing a nitrogen‐based linker such as **13**, chiral information on the palladium center was sufficient to achieve good enantioselectivity (Scheme 4, bottom). The best ligand was found to be phosphoramidite **14**, which allowed differently substituted arylcyclobutanones **15**–**17** to be accessed.

In 2019, Zhang, Dong, and co‐workers showed with a single example that a similar dual activation strategy was also viable for reductive Heck reactions to access formally α‐alkylated cyclobutanones.^[19]^

## 3 Rearrangement

Whereas ring contractions of cyclobutanones are energetically uphill processes, the opposite is true for ring expansions. The release of the inherent ring strain makes such an endeavor particularly favorable, and cyclobutanones are privileged structures for a range of ring extensions.

**Scheme 5:**
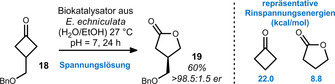
Biocatalytic desymmetrization of cyclobutanone **18** according to Furstoss. Bn=benzyl.

For example, Furstoss and co‐workers relied on enzymes from the fungus *E. echinulata* to conduct an asymmetric Baeyer–Villiger^[20]^ reaction of cyclobutanone **18** (Scheme 5).[^21^, ^22^, ^23^, ^24^] The authors then employed the corresponding lactone **19** in an enantiodivergent synthesis of (*S*)‐ and (*R*)‐proline.

Interestingly, rearrangements of this type are not limited to enzymes, and the upcoming section will focus on non‐enzymatic methods. Since rearrangements are generally initiated upon orbital alignment, precise control of a substrate's conformation is key for achieving selectivity, and many solutions have been inspired by nature. The following section gives an overview of non‐enzymatic transformations.

### 3.1 Baeyer–Villiger Oxidation

**Scheme 6:**
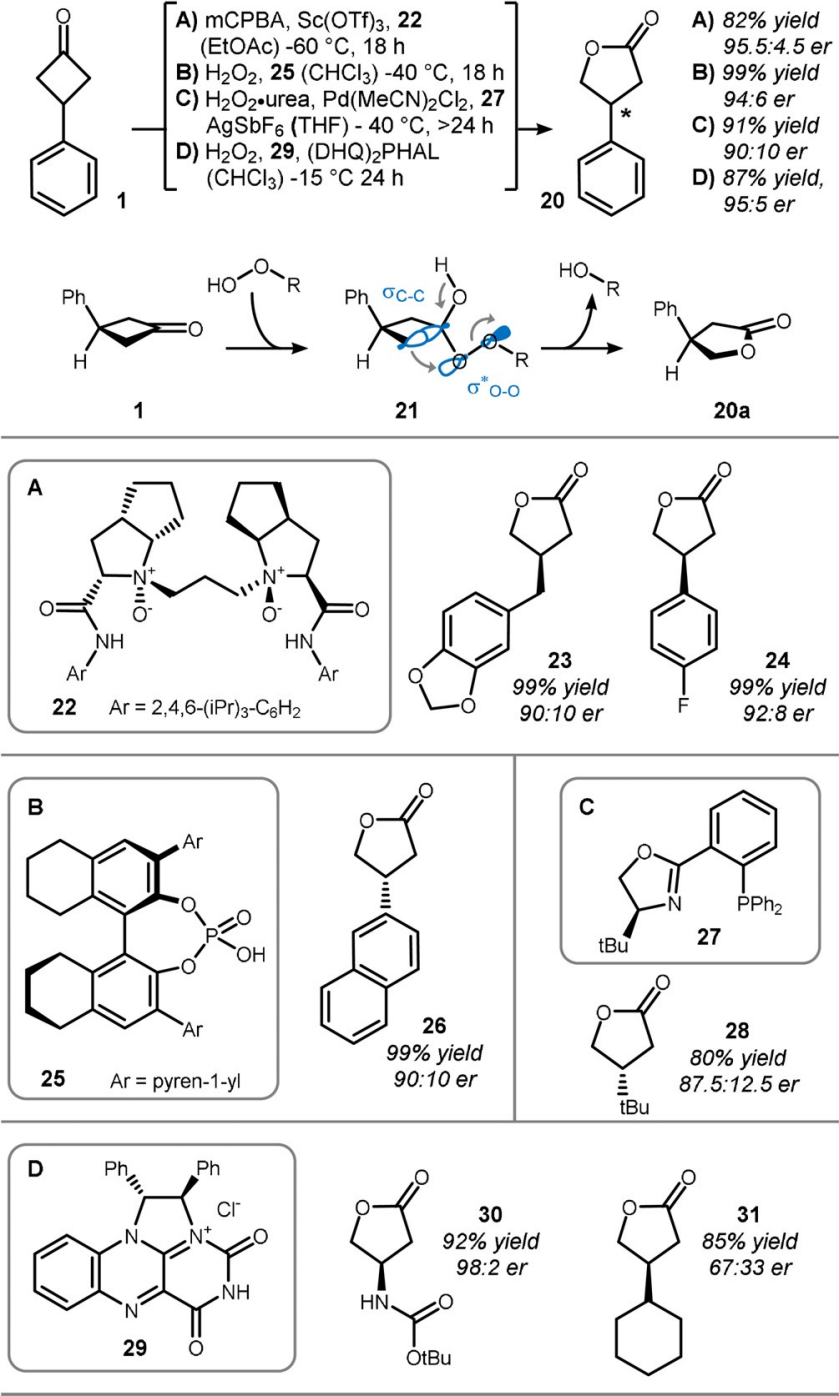
Stereoelectronic rationale behind the Baeyer–Villiger oxidation and recent examples of enantioinduction: A) Lewis acid catalyzed approach of Feng; B) organocatalyzed method of Ding using chiral phosphoric acids; C) transition‐metal‐catalyzed system of Stoltz; D) method of Yamamoto using a self‐assembled flavinium‐(DHQ)_2_PHAL ion pair. mCPBA=*meta*‐chloroperoxybenzoic acid, (DHQ)_2_PHAL=hydroquinine 1,4‐phthalazinediyl diether.

Pioneering studies related to the asymmetric Baeyer–Villiger oxidation of cyclobutanones were conducted by the groups of Lopp, Bolm, and Kotsuki in the late 1990s and early 2000s.^[25]^ As a result of their energetic bias, cyclobutanone desymmetrizations recently became a platform to test new strategies for chiral induction in Baeyer–Villiger‐type reactions. Scheme 6 summarizes recent methods that utilize different types of activation for the reaction of 3‐phenylcyclobutanone (**1**) to lactone **20**.

Mechanistically, a peroxide species adds to the electrophilic carbonyl group to form Criegee intermediate **21**. Based on studies by the groups of Chandrasekhar, Kishi, and Calhoun,^[26]^ rearrangement only happens when the σ‐C−C bond and the σ‐O−O bond are aligned in an antiperiplanar fashion, thereby providing numerous possibilities for catalyst design. Feng and co‐workers developed a Lewis acid based approach, in which chiral ligand **22** induces the respective enantioselectivity.[^27^, ^28^] Structurally diverse lactones such as **23** and **24** were accessible by this sequence in ≥90:10 er (Scheme 6 A). Ding and co‐workers used BINOL‐derived phosphoric acid **25** as a bulky organocatalyst for their enantioinduction (Scheme 6 B).^[29]^ Interestingly, hydrogen peroxide was reactive enough to furnish products such as naphtholactone **26** in good yield and selectivity. Cationic palladium bound to chiral PHOX ligand **27** is also viable, thereby allowing the assembly of alkyl lactones such as **28** (Scheme 6 C, Peterson and Stoltz).[^30^, ^31^]

Yamamoto and co‐workers discovered that flavinium **29** self‐assembles with (DHQ)_2_PHAL to allow the enantioselective formation of carbamate **30** and aliphatic lactone **31** (Scheme 6 D).[^32^, ^33^] The mechanism is most likely related to enzymatic processes, involving an initial addition of hydrogen peroxide to flavinium **29** from where it is then transferred to cyclobutanone **1**.^[34]^

**Scheme 7:**
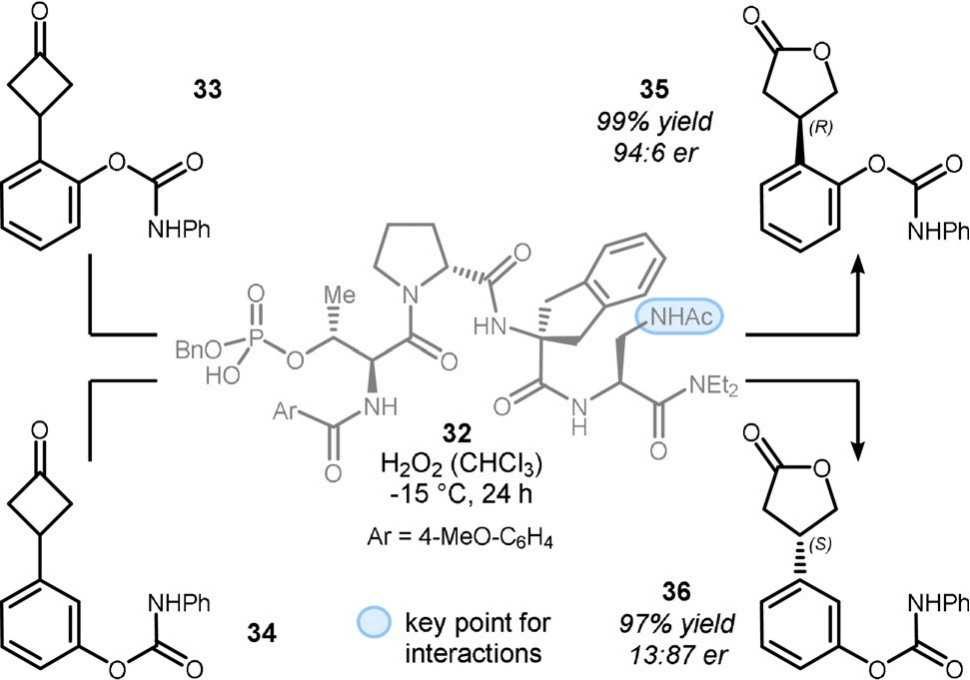
Miller's multifunctional peptide catalyst for the asymmetric Baeyer–Villiger oxidation.

Small peptides with an embedded phosphothreonine unit are also capable catalysts for asymmetric Baeyer–Villiger oxidations. Their mode of action is closely related to BINOL‐derived phosphoric acid **25**, but offers multiple sides for contact with substrates through hydrogen bonding, thus mimicking enzymatic processes. In 2019, Miller and co‐workers introduced oligopeptide **32** as a competent catalyst for such an endeavor (Scheme 7).^[35]^ Depending on the position of the carbamate within the phenyl ring, substrates **33** and **34** underwent Baeyer–Villiger oxidation with reversal of the absolute configuration. Thus, *R*‐configured lactam **35** and *S*‐configured lactam **36** were accessible in excellent yield and good enantioselectivity with the same catalyst **32**. On the basis of structure–selectivity studies, the authors propose hydrogen bonding between the acyl‐protected amine and the polar carbamate group to be crucial for this reversal. Thus, inherent structural biases were overridden and opportunities provided for the functionalization of more complex molecules in the future.

### 3.2 Schmidt and Beckmann‐Type Reactions

**Scheme 8:**
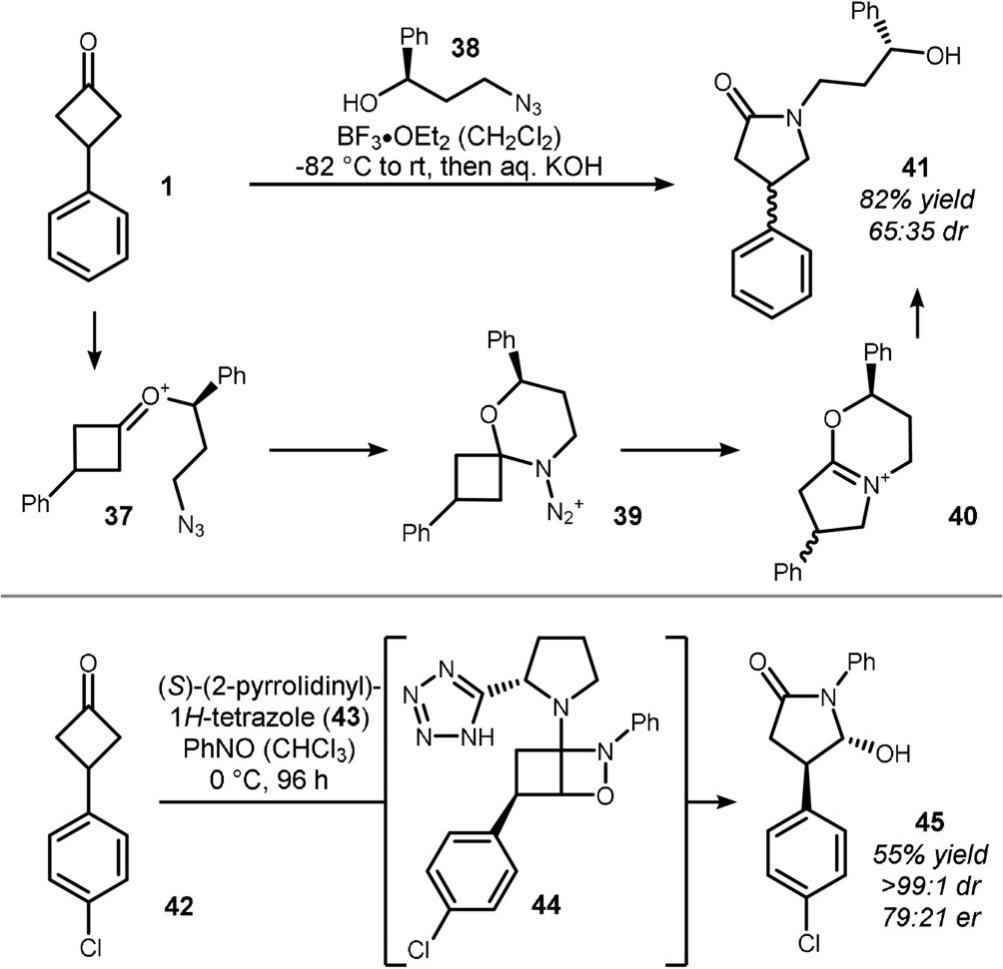
Top: The asymmetric Schmidt reaction developed by Aubé. Bottom: Approach towards enantioenriched lactams used by Piras and Frongia.

The nitrogen analogues of the Baeyer–Villiger oxidation have been less studied in the context of cyclobutanone desymmetrization. Aubé and co‐workers developed an elegant approach for an asymmetric Schmidt reaction through the use of an “in situ tethering” strategy (Scheme 8, top).^[36]^ Boron trifluoride initiates the formation of an oxocarbenium ion **37** from **1** and chiral azidopropanol **38**. This highly electrophilic species is then intramolecularly trapped by the azide to give spirocycle **39**. The chiral information at the six‐membered ring preferentially aligns one of the cyclobutanone's C−C bonds in an antiperiplanar relation to the leaving group. Thus, rearrangement to **40** and subsequent hydrolysis yields lactam **41**. This study primarily focused on cyclohexanone desymmetrization, and a strong correlation between linker length and diastereoselectivity was found during the optimization. Only modest diastereoselectivity was observed for cyclobutanone **1** under these unoptimized conditions. Nevertheless, this investigation represents the first example of the formation of an asymmetric lactam from a cyclobutanone using a cleverly installed linker to initiate the attack of the poorly nucleophilic azide function.

An interesting rearrangement to hydroxylactams was observed by Frongia and co‐workers when they treated 3‐chlorophenylcyclobutanone (**42**) with tetrazole catalyst **43** in the presence of nitrosobenzene (Scheme 8, bottom).^[37]^ Initially, the authors aimed for α‐hydroxylation at the four‐membered ring, as seen in related organocatalytic reactions with unstrained ketones and aldehydes.^[38]^ However, the cyclobutanone iminium collapses prior to hydrolysis and forms the corresponding bicycle **44**, which ultimately reassembles to lactam **45**. The authors were able to prove the structure and absolute configuration of **45** by a formal synthesis of the amino acid baclofen.

## 4 C−C Bond Cleavage

**Figure 1:**
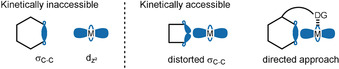
Kinetic barriers for interactions between σ‐C−C bonds and transition metals (M).

In general, C−C bonds are relatively inert to chemical transformations because of their high bond energy and steric inaccessibility. Nevertheless, C−C bonds have the potential to engage in reactions with transition metals under certain conditions and when appropriately activated.^[39]^ The distortion of orbitals through molecular strain and the directing effects of adjacent functional groups enable transition metals to overcome the kinetic barrier and successfully initiate C−C bond cleavage (Figure 1). In addition to the kinetic barrier, thermodynamically favored processes that cleave C−C bonds are rare. One possibility to overcome this issue is through substrate design, as evident by a large number of “spring‐loaded” starting materials and/or intramolecular reactions.

**Scheme 9:**
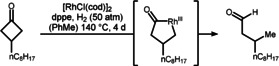
First report of transition‐metal‐catalyzed C−C bond cleavage of cyclobutanones. cod=1,5‐cycloctadiene, dppe=1,2‐bis(diphenylphosphino)ethane.

Cyclobutanones fulfill many of the aforementioned criteria for successful C−C bond cleavage. In addition to the incorporated molecular strain, the α‐C−C bond is further activated by the neighboring carbonyl group. In 1994, Ito and co‐workers were the first to report successful α‐C−C bond cleavage by treating alkyl cyclobutanones with Rh at elevated temperatures (Scheme 9).^[40]^

The prochiral nature of the two α‐C−C bonds in 3‐substituted cyclobutanones provides opportunities for enantioselective desymmetrization. In the past decade, various downstream reactions have been discovered that give rise to a diverse range of complex structures.

### 4.1 Rhodium Catalysis

**Scheme 10:**
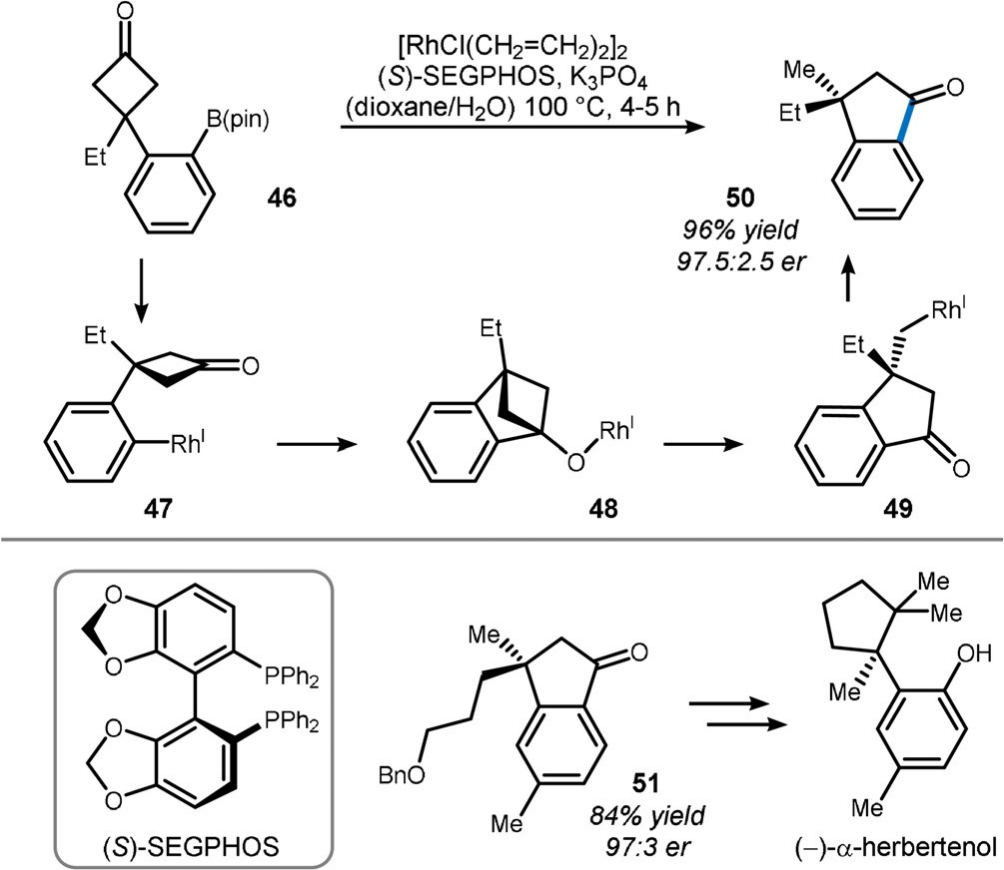
Rh^I^‐catalyzed desymmetrization of arylcyclobutanone boronic esters according to Murakami.

Historically, rhodium was not only the first, but is also the most important transition metal for the cleavage of cyclobutanone C−C bonds.^[41]^ In 2006, Murakami and co‐workers reported an enantioselective Rh‐catalyzed cyclobutanone desymmetrization in which SEGPHOS was utilized as the source of the enantioinduction (Scheme 10).^[42]^ Initially, the transmetalation of boryl cyclobutanone **46** to rhodium delivers phenyl‐rhodium species **47**, thereby placing the metal in proximity to the C−C bond of interest. The authors propose C−C bond cleavage to occur through a two‐step process involving the addition of the arylrhodium compound to the carbonyl group (**47→48**) and site‐selective elimination of a β‐carbon atom. Thus, chiral rhodacyclopentanone **49** is formed, which releases the product **50** after protonolysis. The authors were able to use this method to access cyclopentanone **51**, which served as a starting point in an enantioselective synthesis of (−)‐herbertenol with a highly congested quaternary stereocenter.

**Scheme 11:**
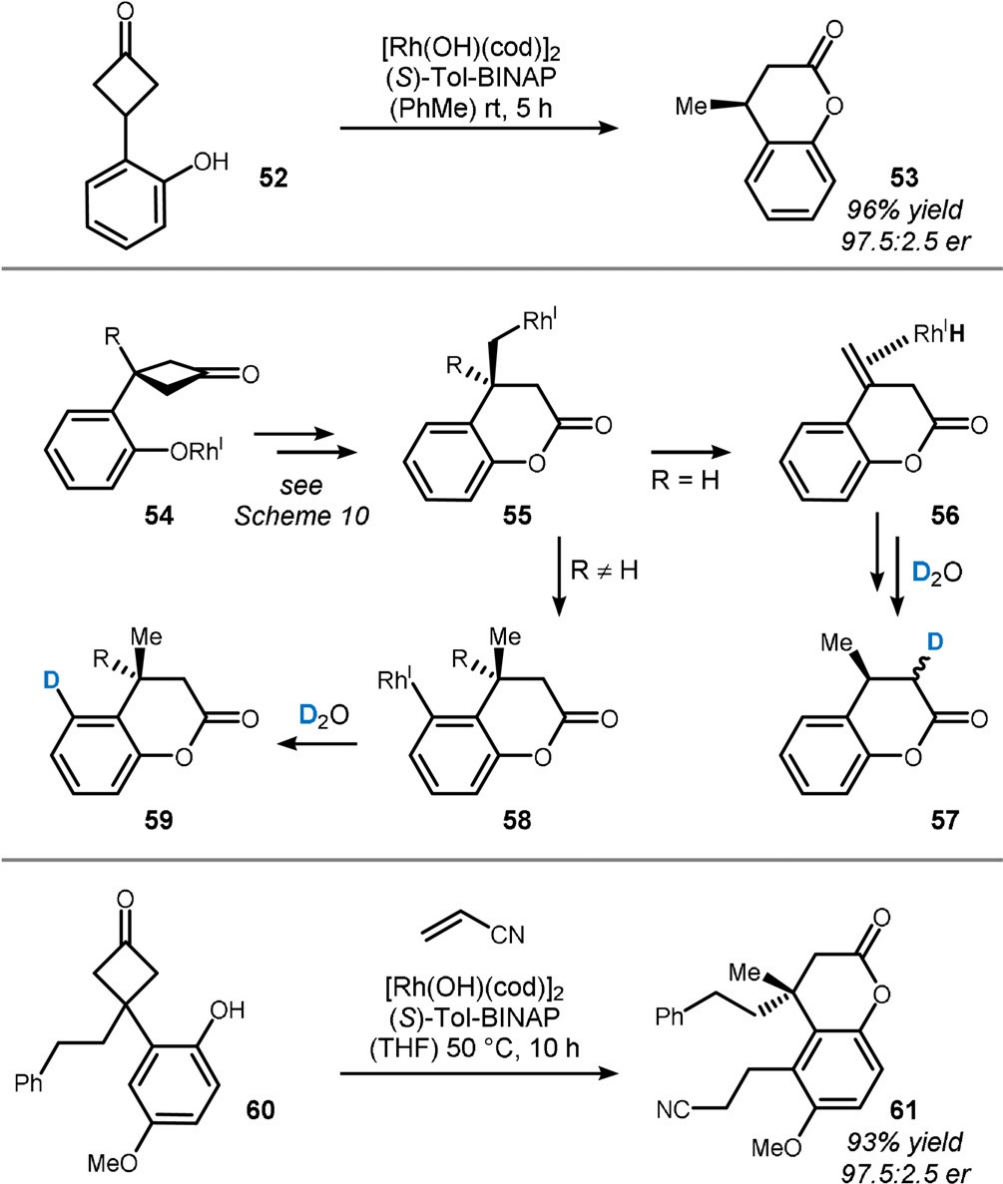
Method used by Murakami to access chiral dihydrocoumarins.

Later, the same group expanded this reaction sequence to hydroxyphenylcyclobutanones such as **52**, thereby enabling the synthesis of dihydrocoumarin **53** and analogues (Scheme 11).^[43]^ The slightly modified BINAP derivative (*S*)‐Tol‐BINAP was found to be a superior candidate for enantioinduction in this case.

Detailed mechanistic studies through deuterium labeling uncovered the following mechanism: Phenoxyrhodium **54** undergoes an addition/β‐carbon elimination sequence that is closely related to that elucidated in Scheme 10 to give rhodadehydrocoumarin **55**.

In the case of R=H, a sequence of β‐hydride elimination/re‐insertion outcompetes protoderhodation and thus enables the rhodium to migrate—notably with high face fidelity—to the thermodynamically favored enolate (sequence via intermediate **56**). A final deuterium abstraction releases product **57**. On the other hand, when R≠H, the rhodium migrates to the aryl group (**55→58**) instead, as indicated by the final position of the deuterium in product **59**. Based on this mechanistic detail, the authors were able to replace the protoderhodation by another migratory insertion through addition of a Michael acceptor. In this remarkable cascade, cyclobutanone **60** was directly converted in 93 % yield and excellent enantioselectivity into polysubstituted coumarin **61** bearing a quaternary stereocenter.

**Scheme 12:**
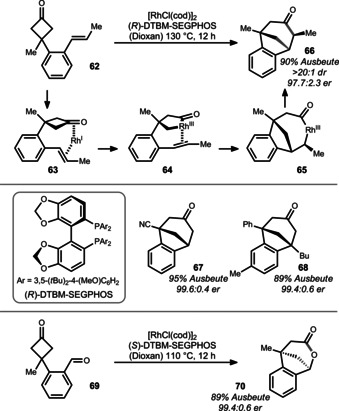
Approach used by Cramer to access benzocycloketones and lactones.

In 2014, Cramer and co‐workers reported a formal C−C bond addition across an alkene (Scheme 12).[^44^, ^45^] Mechanistically, precoordination of rhodium to styrenyl cyclobutanone **62** initiates oxidative addition to one of the enantiotopic C−C bonds of complex **63**. The authors propose that the corresponding rhodacyclopentanone **64** undergoes migratory insertion to give bicyclic rhodacycle **65**, followed by a final reductive elimination.

High stereospecificity together with low epimerization rates allow the synthesis of thermodynamically unfavored **66** in high diastereoselectivity. Consecutive treatment with base allowed epimerization to *epi*‐**66**. The overall high enantioselectivity was secured by DTBM‐SEGPHOS, thereby making optically active nitrile **67** and ketone **68** also readily available.

Interestingly, when aldehyde **69** was subjected to the reaction conditions at 110 °C, a formal alkylacylation was observed and the corresponding lactone **70** isolated.^[46]^ The mechanism likely follows a similar course as the alkenyl case before. Overall, this method is very powerful as it establishes two stereocenters, both (if desired) quaternary, in a single step. In addition, the bicyclic ketone or lactone structures provide plenty of opportunities for further functionalizations.

**Scheme 13:**
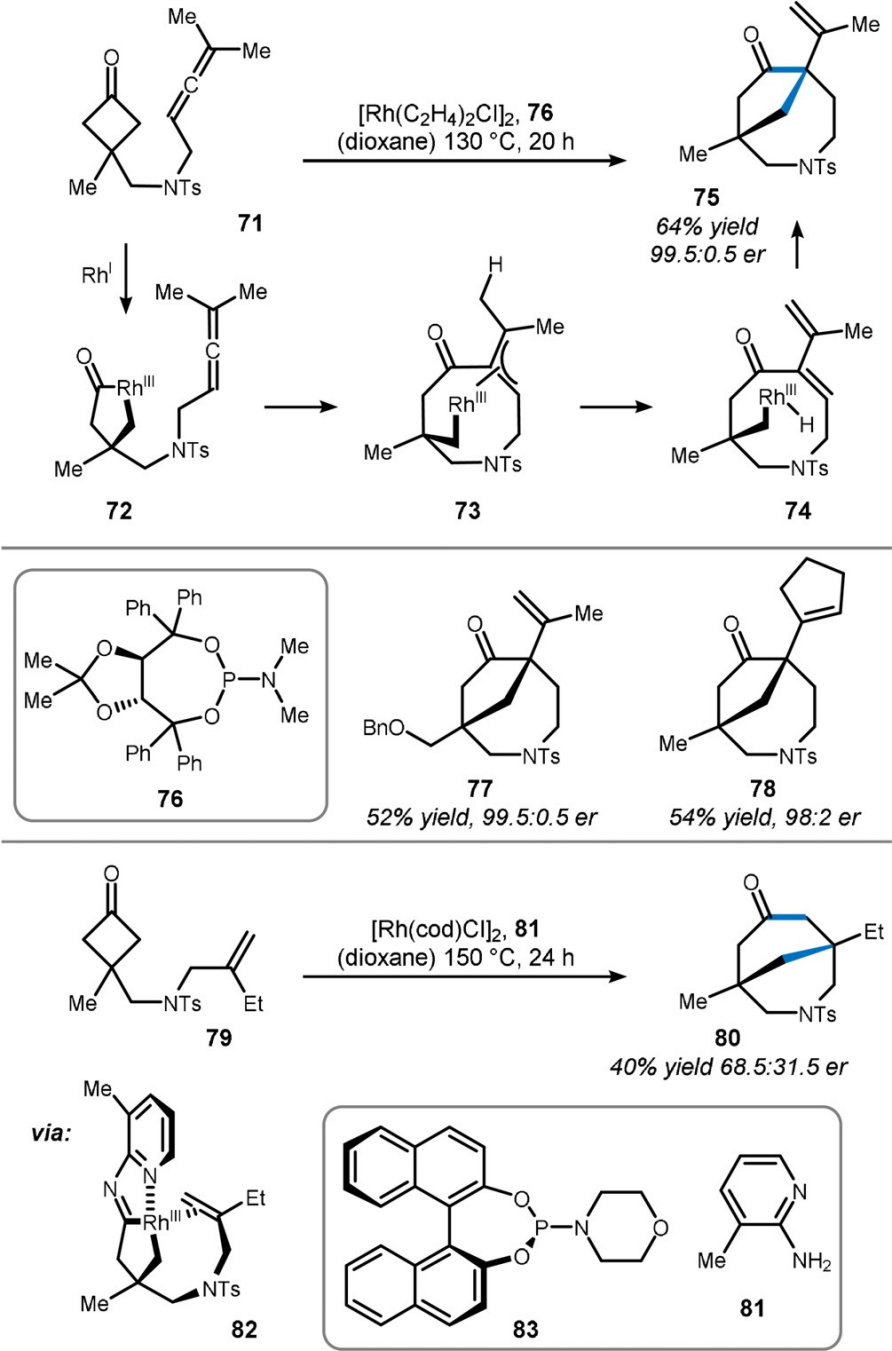
Asymmetric method used by Dong to access bridged ring systems.

In contrast to alkenes, allenes undergo intramolecular insertion into cyclobutanones in a [4+1] fashion (Scheme 13).^[47]^ In their detailed study, Zhou and Dong propose an initial oxidative addition of cyclobutanone **71** to rhodium. Intermediate **72** then forms allylic rhodacycle **73**, which is reluctant to undergo reductive elimination. Instead, β‐hydride elimination gives rhodium hydride **74**, whose existence was corroborated by deuterium labeling of the methyl group. Finally, re‐insertion of either Rh−H or Rh−C followed by reductive elimination releases the [4.2.1] bicyclic structure **75**. The authors also excluded an initial allene isomerization through control experiments. The excellent level of enantioselectivity (>99:1 er) can be ascribed to the addition of phosphoramidite **76** as a ligand. Structurally divergent products such as benzyl ether **77** and cyclopentene **78** were also well‐tolerated in this sequence.

In contrast to allenes, the insertion of unactivated alkenes such as **79** occurs in a [4+2] fashion to provide [3.3.1] bicycle **80**, in close analogy to Cramer's study with styrenyl cyclobutanones. In this study,^[48]^ Ko and Dong relied on the transient directing group 2‐aminopyridine **81** to assist with the oxidative addition.^[49]^ In contrast to the previous examples, inferior precoordination and a relatively flexible alkene linker kinetically hinder the smooth oxidative addition. Thus, intermediate **82** is a likely intermediate en route to azepane **80**. Even though ligand **83** only allowed for moderate enantioselectivity, this reaction represents a rare example of the successful utilization of an unactivated alkene in such a complex setting. In general, this type of [4+2] cyclobutanone–alkene fusion resembles a complementary approach to the intramolecular Diels–Alder‐type disconnection, further highlighting its importance.

### 4.2 Nickel Catalysis

**Scheme 14:**
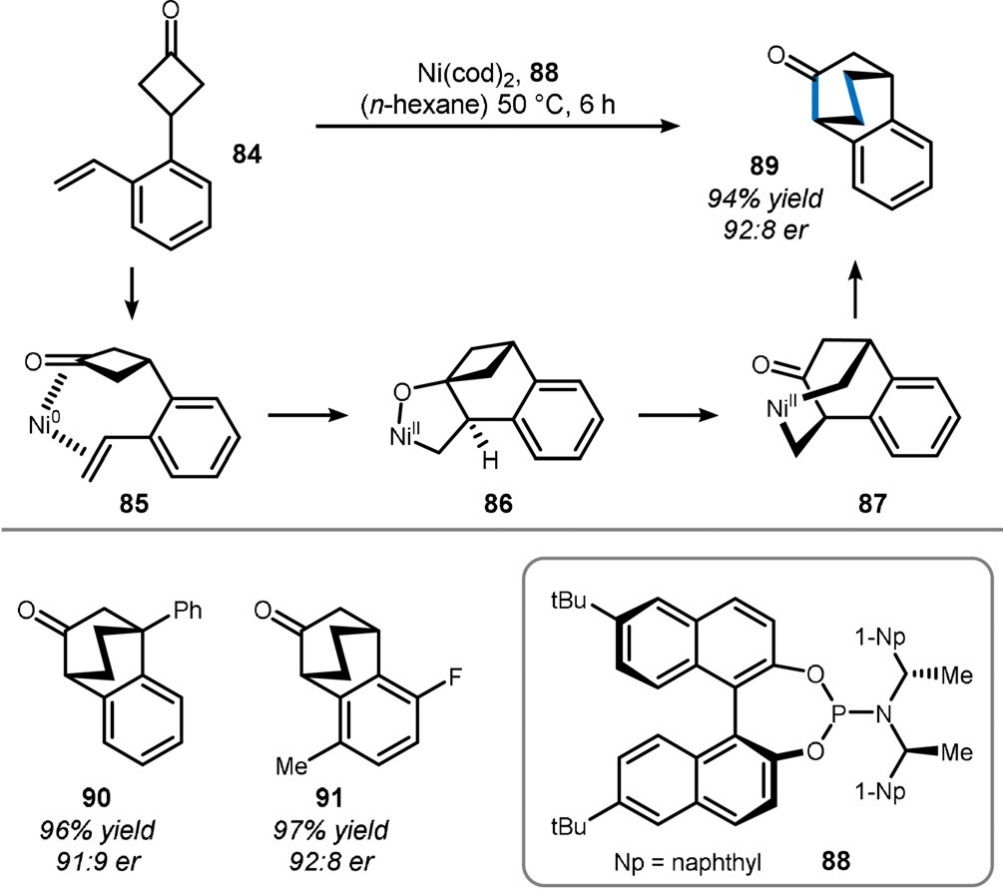
Method used by Murakami to access chiral benzobicyclo[2.2.2]octenones.

Nickel often exhibits complemental reactivity to rhodium and has become important for the enantioselective desymmetrization of cyclobutanones. For example, styrenyl cyclobutanone **84** undergoes a different type of skeletal rearrangement (Scheme 14), as seen previously for rhodium in Scheme 12. The mechanistic rationale behind this divergence is based on metal‐specific preferences for oxidative addition versus oxidative cyclization.[^50^, ^51^] Thus, a plausible mechanism comprises precoordination of nickel(0) to the C=C and C=O π‐bonds (intermediate **85**) triggering oxidative cyclization to oxa‐nickelacycle **86**.^[52]^ It is important to note that this step establishes benzylic stereocenters, which only render selective β‐carbon elimination to **87** possible. In this study by Murakami and co‐workers, phosphoramidite **88** enables selective cyclization by energetically discriminating one of the prochiral faces of the alkene to bind to nickel.

Thus, excellent enantioselectivity was achieved and bicyclo[2.2.2]octenone **89** was isolated in 94 % yield. Quaternary stereocenters (phenyloctenone **90**) as well as substitution at the ring (fluoroarene **91**) were well‐tolerated, thus making this procedure valuable—particularly because of the importance of bicyclo[2.2.2]octanols as calcium channel blockers.^[53]^

**Scheme 15:**
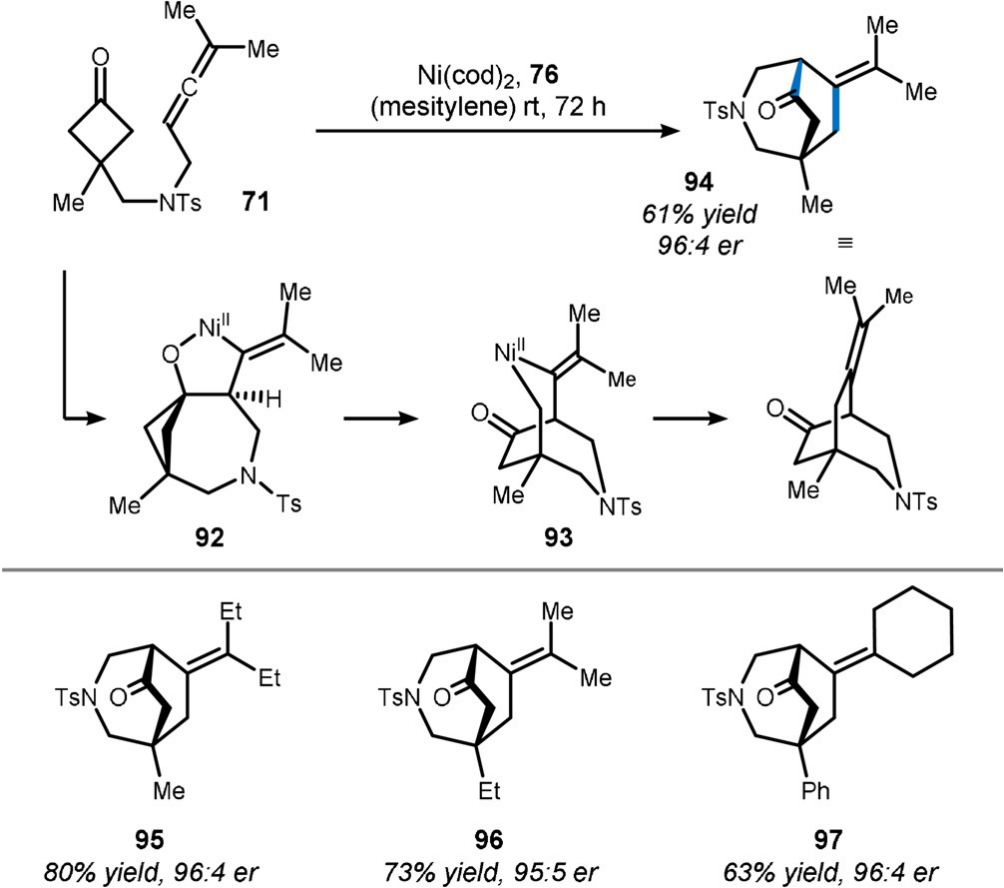
Nickel‐catalyzed desymmetrization using allenes according to Dong.

A similar metal‐dependent regiodivergence was observed by Zhou and Dong when allenyl cyclobutanone **71** was subjected to nickel(0) instead of rhodium (Scheme 15).^[54]^ Here, a sequence of oxidative cyclization (**71**→**92**) and β‐carbon elimination (**92**→**93**) explains the formal [4+2] cyclization to azepane **94** (versus the [4+1] cyclization in Scheme 13). In this study, ligand **76** gave the best enantioselectivity, as illustrated through the differently decorated products **95**, **96**, and **97**. Overall, this example highlights how achiral starting material **71** serves as a precursor for the fast assembly of constitutional isomers **75** and **94** with multiple stereocenters.

### 4.3 Palladium Catalysis

**Scheme 16:**
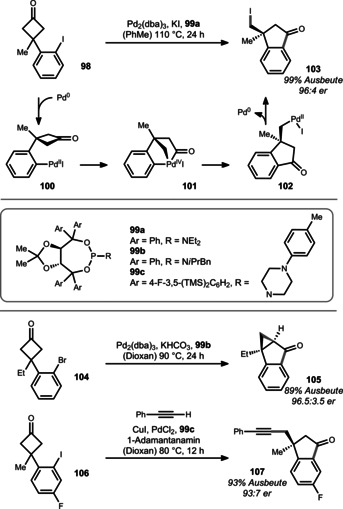
Cascade used by Xu towards benzocyclopentanones bearing a quaternary stereocenter. dba=dibenzylidene, TMS=trimethylsilyl.

Given the plethora of data on palladium‐catalyzed C−C bond formation,^[55]^ it is surprising how little is known about C−C bond cleavage, specifically in the context of cyclobutanones.^[56]^ Recently, the Xu group developed an enantioselective σ‐bond reshuffling for cyclobutanones (Scheme 16; e.g. using cyclobutanone **98**).^[57]^ Enantioinduction was provided in this reaction by TADDOL‐derived phosphoramidite **99 a**. Mechanistically, the authors originally proposed a reaction sequence closely related to the rhodium example in Scheme 10.^[56a]^ DFT calculations suggest an alternative mechanism in which all the common oxidation states of palladium are energetically favored.^[56b]^ Herein, aryl iodide **98** oxidatively adds to Pd^0^ to form arylpalladium iodide **100**.

Oxidative addition to the α‐C−C bond of the cyclobutanone subunit leads to palladium(IV) species **101**, which subsequently undergoes reductive elimination to cyclopentanone **102**. The neopentylic palladium species is prone to a second reductive elimination to form the C−I bond of product **103**. The authors found that the same product **103** could also be accessed from an aryl bromide substrate by addition of potassium iodide through an in situ bromine–iodine exchange. When no iodide was present (e.g. with bromide **104** and ligand **99 b**), reductive elimination occurred from an intramolecular carbon‐bound enolate to furnish cyclopropane **105** in excellent yield. Interestingly, intermediate **102** could also undergo Suzuki‐ and Sonogashira‐type coupling when the appropriate nucleophile was added and a slightly modified ligand used. An illustrative reaction is the conversion of fluoroaryl cyclobutanone **106** into cyclopentanone **107** using ligand **99 c**, which establishes a quaternary stereocenter with good efficiency. The flexibility of palladium to allow the formation of different products is remarkable and highlights its ability for divergent reaction development.

## 5 Conclusions

Cyclobutanones are unique building blocks that allow a variety of unusual reactions and the generation of vast molecular complexity. The combination of the overall downhill energy profile and enantioselective desymmetrization enables multiple stereocenters to be accessed in a single operation. The asymmetric functionalization of cyclobutanones provides rigid 3D structures that are interesting as potential drug candidates in medicinal chemistry. Whereas the Baeyer–Villiger rearrangement has been studied extensively, asymmetric versions of the nitrogen and carbon analogues are mostly unknown. The recent introduction of alternative transition metals for C−C bond cleavage opens new avenues for divergent syntheses, and many metals have never been studied in this context. The same holds true for β‐C−C bond breaking and ring‐opening reactions, which could also be powerful approaches. Inspiration for synthetic endeavors, especially in terms of total synthesis, might be worth considering, since breaking the symmetry enables the sophisticated and atom‐economic construction of molecular frameworks.

## Conflict of interest

The authors declare no conflict of interest.

## Biographical Information

*Jan Sietmann obtained his B.Sc. in chemistry from the Westfälische Wilhelms‐Universität Münster (2016). During his Master studies in 2017 he worked at Armacell GmbH, where he focused on the storage stability of adhesives. He completed his M.Sc. on the synthesis of a ganglioside in the group of Prof. Ryan Gilmour in Münster in 2018. He is currently a graduate student in the group of Dr. Johannes Wahl, where he is working on the enantioselective ring expansion of cyclobutanones*.

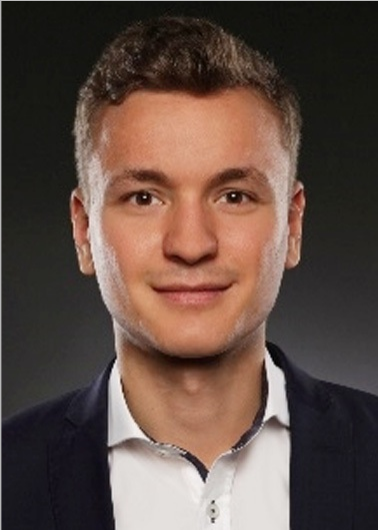

## Biographical Information

*Johannes M. Wahl obtained his M.Sc. in 2012 from the University of Basel*, *Switzerland. After research with Prof. Donna G. Blackmond at The Scripps Research Institute, he moved to Technische Universität München to pursue his Ph.D. with Prof. Thorsten Bach. After obtaining his Ph.D. in 2016 and a subsequent postdoctoral appointment with Prof. M. Kevin Brown at Indiana University, he started his independent career as a junior research group leader at the Westfälische Wilhelms‐Universität Münster in 2019. His research focuses on strain‐mediated reaction development and application in natural product synthesis*.